# Getting to the edge: protein dynamical networks as a new frontier in plant–microbe interactions

**DOI:** 10.3389/fpls.2014.00312

**Published:** 2014-06-30

**Authors:** Cassandra C. Garbutt, Purushotham V. Bangalore, Pegah Kannar, M. S. Mukhtar

**Affiliations:** ^1^Department of Biology, The University of Alabama at BirminghamBirmingham, AL, USA; ^2^Department of Computer and Information Sciences, The University of Alabama at BirminghamBirmingham, AL, USA; ^3^Nutrition Obesity Research Center, The University of Alabama at BirminghamBirmingham, AL, USA

**Keywords:** systems biology, plant–pathogen interactions, regulatory network, protein–protein interactions, functional modules, network dynamics, edgetics

## Abstract

A systems perspective on diverse phenotypes, mechanisms of infection, and responses to environmental stresses can lead to considerable advances in agriculture and medicine. A significant promise of systems biology within plants is the development of disease-resistant crop varieties, which would maximize yield output for food, clothing, building materials, and biofuel production. A systems or “-omics” perspective frames the next frontier in the search for enhanced knowledge of plant network biology. The functional understanding of network structure and dynamics is vital to expanding our knowledge of how the intercellular communication processes are executed. This review article will systematically discuss various levels of organization of systems biology beginning with the building blocks termed “-omes” and ending with complex transcriptional and protein–protein interaction networks. We will also highlight the prevailing computational modeling approaches of biological regulatory network dynamics. The latest developments in the “-omics” approach will be reviewed and discussed to underline and highlight novel technologies and research directions in plant network biology.

## SYSTEMS BIOLOGY: A PARADIGM SHIFT FROM REDUCTIONISM

**FIGURE 1:**
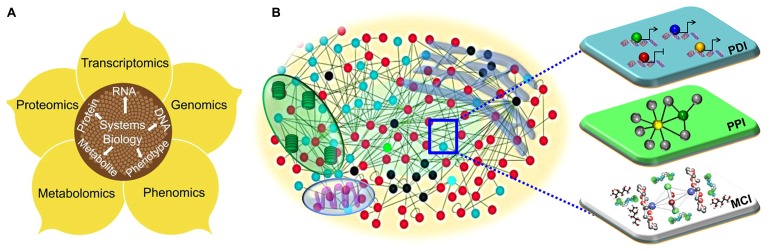
**The systems biology approaches to understand plant immune systems. (A)** The diagrammatic overview of the integrative framework of multiple layers of “-omics” including genomics, transcriptomics, proteomics and phonemics. **(B)** Visualization of a cell as a complex web of macromolecular interactions that constitutes an “interactome.” Functional modules, such as transcriptional (protein–DNA interactions; PDI), translational (protein–protein interactions; PPI) and metabolic (metabolite–compound interactions; MCI) are illustrated.

Despite the progress of understanding phytopathogenic microbes and plant infectious diseases, the arms race between hosts and pathogens fuels further scientific research (Boyd et al., 2013). Within the past decades, the molecular approaches to solve these crises entailed reductionism that seeks to explain a biological system through the summation of its isolated parts. While conceptual origins of systems biology date back almost 100 years, a shift from the reductionist approach to a more inclusive and integrative one started to occur at dawn of this millennium (**Figure 1A**; Arkin and Schaffer, 2011). This revolutionary, holistic approach is inspired by Aristotle’s belief that “The whole is more than the sum of its parts.” “Systems” has also been referenced as the “fifth fundamental requirement for Life” considering that biological structures and molecules never function in isolation, as is true for sociological structures (Carvunis et al., 2013). The limitations of reductionism with respect to medical science are widely recognized and systems biology offers a way of transcendence (Ahn et al., 2006). Extending this observation further, the tenets of systems biology certainly offer an alternative viewpoint for other biological research including plant biology. In addition, this holistic approach can be attributed to the scientific community’s search for understanding the complexity and interconnectedness in a wide array of natural systems ranging from the microscale of a cell to the macroscale of socioecosystems. It has become strikingly evident that significant similarities exist at the structural organization levels among the extremes of these biological spectra (Keurentjes et al., 2011). Thus, systems biology yields models that analyze various changes in biological systems over time, and a systems perspective complements reductionism to facilitate innovative investigations and discoveries (Mitra et al., 2013). It also seeks to uncover the unpredictable and predictable intricacies of many different causal relations within diverse biological components. Cumulatively, a systems approach to medical and agricultural research could guide new developments in techniques, knowledge, and ultimately therapeutics (Barabasi et al., 2011; Vidal et al., 2011).

Here, we will review various elements of systems biology beginning with the level of the “-omes” (**Figure 1A**) and then elaborate on the scale of macromolecules and their interactions. The application and translation of any discovery teems with possibilities from improvements in medicinal therapeutics and plant biology to improvements in crop yield, quality, and pathogen resistance.

## THE CELL AS A COMPLEX WEB OF MACROMOLECULAR INTERACTIONS

In any eukaryotic cell, thousands of genes and their products orchestrate their transcriptional, translational, and metabolic activities to create cellular functions, phenotypic plasticity and organismal fecundity. Functional modules embedded within protein–DNA interactions, and protein–protein, and metabolite–substrate networks execute diverse cellular functions (**Figure 1B**; Mitra et al., 2013). The dichotomous (deterministic or stochastic) nature of network modules is beneficial to cells or organisms for adaptation to physiological perturbations, environmental cues, or pathological signals (Shmulevich and Aitchison, 2009). On the contrary, pathogens have evolved a suite of virulence proteins (effector molecules) that perturb the intracellular networks of their hosts to cause infection (Mukhtar, 2013). As with any host–pathogen conflict, plants and their pathogens are in an evolutionary “arms race,” in which the host mounts defenses, the pathogen develops new strategies to thwart the defensive mechanisms, which in turn forces the host to adapt (Mukhtar et al., 2011; Pajerowska-Mukhtar et al., 2013). Network-based analysis is a holistic approach that can enable a detailed understanding of the relationships between phytopathogens and plants (Pritchard and Birch, 2011).

**FIGURE 2:**
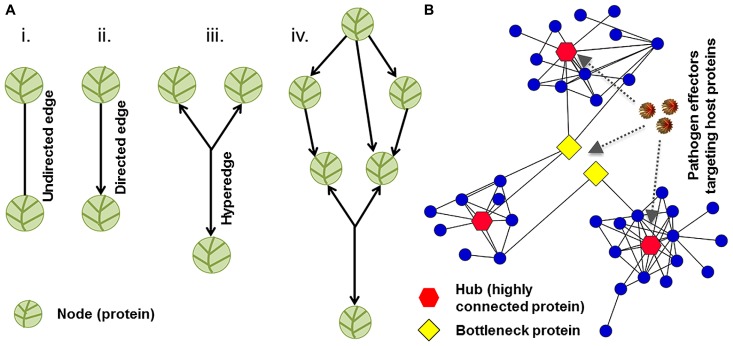
**Network structure and topology. (A)** The organization of nodes and edges in a graph represents network structure. Vertices and links represent nodes and edges, respectively. Two nodes can be connected by undirected or directed edges. **(B)** A sub-network of plant–pathogen interactions is demonstrated. Hubs (highly connected proteins), bottlenecks (high betweenness nodes), and pathogen effectors (virulence factors) are depicted in red, yellow, and brown colors, respectively. Network with scale-free topology might be vulnerable to pathogen-mediated perturbations.

Network biology, a branch of systems biology, translates the complexities of molecular interactions into a biological message. Any given genotype has a sophisticated underlying network of macromolecular interactions that give rise to a phenotype. The idea behind systems biology is that cellular networks and biological systems are the bridges from genotype to phenotype (Carvunis et al., 2013). Typically in a network, physical and functional interactions between molecules are referred to as edges, and the molecules involved in the interactions are termed nodes. Nodes can correspond to nucleic acids, proteins, hormones, metabolites, or other macromolecules. Edges can be directed or undirected depending on the type of an interaction being illustrated in the graph (**Figure 2A**, Seebacher and Gavin, 2011). Computational biologists and mathematicians have developed numerous algorithms to analyze the versatile relationships of nodes and understand the cellular organization of communication for a particular network. Research efforts to uncover potential universal laws that govern cellular networks are underway (Carvunis et al., 2013).

## DYNAMICS OF TRANSCRIPTIONAL REGULATORY NETWORKS IN PLANT DEFENSE

Upon pathogen recognition, the plant cell undergoes an extensive transcriptional reprogramming in a highly dynamic and temporally regulated manner. Stimulation of these plant defenses involves complex signal transduction networks incorporating feedback and cross-talk controlled by largely unknown mechanisms (Mukhtar et al., 2009). While transcriptomics has already uncovered hundreds of pathogen-responsive genes and key regulatory nodes, a large-scale immune transcriptional regulatory network is yet to be generated. In building a transcriptome, yeast-one-hybrid, and chromatin immunoprecipitation assays are especially useful as they can differentiate between indirect and direct gene regulation (Vidal et al., 2011). Experimental large-scale and static cellular networks give insight into biological systems at certain times and conditions. This data is often combined with protein localization data, protein–protein interactions (PPIs) and other temporal expression data (Uzoma and Zhu, 2013). Network dynamics modeling enables changes in transcriptional networks, interactomes, and signaling pathways to connect genotypic changes with plant defenses and disease phenotypes. Furthermore, immune-related subnetworks or modules help decode the complexities within biological systems (Riccione et al., 2012). By analyzing network dynamics from an evolutionary perspective, a phylogenetic relationship among molecules can also be identified (Morange, 2013; Soyer and O’Malley, 2013). Here, we explore a number of existing computational tools and algorithms that can be exploited to predict, model and determine the dynamics of plant immune regulatory networks.

Static plant transcriptional immune networks are usually inferred using both linear and non-linear correlations as well as non-linear dimensionality reduction method (RepEdLEGG; Ernst et al., 2007; Sato et al., 2010). RepEdLEGG was employed on transcriptomic data obtained from diverse *Arabidopsis* immune mutants to model a static immune signaling network (Sato et al., 2010). Dynamic regulatory events miner (DREM) utilizes an input–output hidden Markov model and gene expression time series data to construct dynamic regulatory networks (Schulz et al., 2012). Recently, DREM was used to analyze ethylene transcriptional response in context of dynamic EIN3 binding data (Chang et al., 2013). Signaling and DREM (SDREM) extends DREM to address perturbation in the regulatory networks (Gitter et al., 2013). However, modeling dynamic interactions of the genes and generating meaningful perturbations requires a more expanded framework that must incorporate dynamic data as well as any environmental dependencies. To consider solutions available to address dynamic network perturbations, Shannon’s mutual information was used to model dynamic relationships of genes and show that both linear and non-linear models could be incorporated while integrating dynamic and environment-dependent complexities of gene expression (Wang et al., 2013). NEXCADE is another interactive network perturbation program, that uses a graph theoretical approach and simulates single, multiple, and sequential perturbations (Yadav and Babu, 2012). However, it remains to be determined whether these algorithms are effective in modeling plant immune system network. Furthermore, standardized qualitative dynamical modeling suite (SQUAD) uses a binary decision diagram algorithm to identify all the stable steady states and then applies a qualitative dynamical systems approach to solve the resulting continuous dynamic system (Di Cara et al., 2007). Noteworthy, Naseem et al. (2012) recently utilized SQUAD to perform dynamic modeling of the plant hormonal signaling network.

Computational cost is another essential aspect in modeling large-scale dynamic regulatory networks. Such cost can be drastically reduced by using software equipped with a deterministic model along with a heuristic algorithm, such as NetGenerator V2.0 (Weber et al., 2013). Collectively, in the light of aforementioned bioinformatics tools, an expanded computational framework is needed that incorporates expression data with multiple timescales, cellular compartments, host proteins–pathogen effector interactions and other environmental dependencies to model plant–pathogen interactions networks.

## HOST–PATHOGEN PROTEIN–PROTEIN INTERACTION NETWORKS

Complementary proteomics analyses are essential to understand global virulence effects caused by pathogens’ effector-mediated perturbations of the key nodes in the plant immune system. The first plant–pathogen interaction network-1 (PPIN-1) was constructed using effectors from two pathogens spanning the eukaryote–eubacteria divergence and three classes of *Arabidopsis* immune system proteins (Mukhtar et al., 2011). The resulting network contains 3,148 interactions among 926 proteins. The PPIN-1 also identified 165 effector-interacting proteins (effector targets), compared to only approximately 20 described previously. While a stringent yeast-two-hybrid (Y2H) system was employed for the above analyses, the common limitations of this heterologous system may still apply concerning both the false-positive and false-negative discovery rates. In addition, PPIN-1 revealed that pathogen effectors target highly interconnected host machinery to suppress effective host defenses and promote pathogen fitness (**Figure 2B**; Mukhtar et al., 2011). Several network biology hypotheses/premises have been developed through interactome mapping. The centrality–lethality rule and local impact hypothesis are two examples that have been applied to human diseases (Arabidopsis Interactome Mapping Consortium, 2011; Barabasi et al., 2011; Gulbahce et al., 2012). According to the centrality–lethality rule, nodes that are central to many connections have the potential of dismantling the entire system if disabled, such as through a viral attack. The local impact hypothesis states that “products of disease susceptibility genes should reside in the network vicinity of the corresponding viral targets” (Gulbahce et al., 2012). For this study, the host interactome was developed by integrating different data sources. Epstein–Barr virus and human papillomavirus strains were selected to explore mechanisms of virally implicated diseases. These strains were found to target host proteins that were in proximity to other proteins associated with viral diseases as evidenced by significant shift in gene expression levels in corresponding disease implicated tissues (Gulbahce et al., 2012). Viral “neighborhoods” existed in the host interactome and were labeled as “viral disease networks.” Similar demonstrations or contradictions need to be studied in the plant kingdom. Considering network components, hubs (highly connected proteins) and edges play an integral role in human and *Arabidopsis* immune systems. In humans, understanding the role of hub proteins requires differentiating between disease-related genes and essential genes. Given that human hubs frequently correspond to disease-related proteins (Barabasi et al., 2011), it would be interesting to extend this theory to plants for testing and verification in diverse natural populations. While several high-throughput technologies have been applied in plants, there still exist hundreds of thousands of unconstructed plant cell PPIs. Construction and access to reference PPIs can be achieved through computational and predictive methods. Predicative capabilities are based on a wide range of protein and interactome characteristics (Fukunishi and Nakamura, 2008; Lee et al., 2010). The Protein Data Bank serves as a reference for proteins’ three-dimensional structures and protein complexes (Velankar and Kleywegt, 2011; Velankar et al., 2012; Gutmanas et al., 2014). As a predictive tool, the Protein Data Bank is a methodological starting point for exploring experimentally determined protein interfaces, emphasizing particular features that can be used to predict domain–domain interactions in proteomes (Braun et al., 2013). Protein docking software provides another category of methods to infer protein–protein binding domains and interaction sites (Cai et al., 2013; Pencheva et al., 2013).

## DIFFERENTIAL AND THREE DIMENSIONAL NETWORKS

While the development of comprehensive reference maps is one of the current challenges in the field, the future of omics-based research will integrate biological insights into networks to drive translational research. Creating comprehensive reference network maps is the first step toward developing dynamic, information-rich resources. To assist in these efforts, standardized experimental benchmarking and validation assays provide a mechanism to estimate the size and validity of the existing networks (Braun et al., 2013). In contrast to the highly dynamic and fluctuating endogenous conditions, under which biological systems normally operate, most physical interactome maps are developed from experiments conducted under static conditions. Differential network mapping takes into account the dynamic state to produce a cell-type and condition-specific interactome (Carvunis et al., 2013). Due to this characteristic, differential network mapping provides a more accurate description of the molecular and cellular mechanisms within a living system. Although network biology provides a platform for decoding complexity, collapsing of networks to nodes and edges may lead to a significant loss of data. Current two-dimensional interactome maps do not consider either structure or conformation of the individual proteins within a network and ignore the spatial limitations of protein interactions. Because protein structure and function are highly interwoven, three-dimensional interactome maps that account for protein structure, interfaces, and even isoforms can greatly enhance the level of understanding of *in vivo* PPIs (Stein et al., 2011; Wang et al., 2012; Zhang et al., 2012).

Two-dimensional protein networks can be reconstructed with a third dimension to integrate protein structure, conformation, and spatial limitations. Atomic-level protein structure information was resolved for several large-scale human PPI networks to create the third dimension of analysis (Das et al., 2014a, b). Previously, a three-dimensional reconstruction of protein networks was conducted to elucidate the genetic and molecular mechanisms underlying human diseases; this investigation primarily focused on gene pleiotropy and locus heterogeneity (Wang et al., 2012). This type of network construction could also be applied to plant network maps in an effort to better understand plant disease and genotype–phenotype complexity. To assist with the creation of 3D interactome networks, the first iteration of interactome networks with structural information (INstruct; a database that houses current high quality, three-dimensional PPI networks that are structurally resolved to the atomic level) was built using several model organisms (Meyer et al., 2013). INstruct includes 37 *Schizosaccharomyces pombe*, 1273 *Saccharomyces cerevisiae*, 119 *Mus musculus*, 166 *Drosophila melanogaster*, 120 *Caenorhabditis elegans*, 644 *Arabidopsis thaliana*, and 6585 human interactions.

## NODE AND EDGETIC INVESTIGATIONS

Network components and topological properties provide novel avenues of investigation. Phenotypic variations due to total loss of a gene product (node-removal) emphasize the importance of node-centered investigations. Network topological properties of a node can be investigated to determine key proteins that are central to many interactions (Barzel and Barabasi, 2013). One topological property involves the degree of a node, which describes the number of edges a node has within a network. Hubs are central and critical to many edges within a network. In a scale-free network, most nodes possess few connections to other nodes while a handful of hubs essentially form the foundation of the network. This characteristic of scale-free networks is incorporated in PPI and metabolic network maps developed for organisms ranging from yeast to humans (Vidal et al., 2011). Recently, several independent studies confirmed the importance of hub proteins in pathogen virulence mechanisms. The results indicate that diverse pathogen proteins (spanning across viruses, bacteria, and fungi) target hub proteins in both humans and plants (Vidal et al., 2011; Braun et al., 2013). Thus far, two different categories of network hubs have been identified. Party and date hubs differ by their number of edges and the conditions that enable the interaction (Vidal et al., 2011). Party hubs are known for maintaining connections with all of their partners in all tested conditions. Date hubs tend to interact with different partners based on specific conditions. Node-removal can affect inter- and intra-network hub proteins or ensue on the periphery of a community of proteins. Other phenotypic variants can arise from edgetic perturbations (removal of a specific edge). In an edgetic disruption, a targeted interaction is disrupted while all other interactions (edges) remain unaffected. The consequence of node-removal on the structure of the network might be greater because removing a node impacts more than one specific interaction (Zhong et al., 2009). Conversely, edgetic perturbations produce less significant network structure changes. At the molecular level, edgetic disruptions are characterized by in-frame point mutations that cause single amino acid substitutions and minute insertions, whereas truncating mutations and deletions reflect node-removal mechanisms. Given that about half of the ~50,000 known human diseases could be linked to edgetic disruptions (Zhong et al., 2009; Vidal et al., 2011), a similar application of the edgetic hypothesis to the plant kingdom can potentially shed light on disease and abiotic stress responses, yielding tools for crop improvement.

New methods such as forward and reverse edgetics aid in the analysis of phenotypic variation due to disturbances in specific molecular interactions (Charloteaux et al., 2011). Forward and reverse edgetics are complementary strategies of phenotypic investigation. Forward edgetics takes a mutated gene associated with a specific phenotype and uses Y2H to establish the interaction disruption. Reverse edgetics begins with a protein of interest and its corresponding set of interactions. Using reverse Y2H screens, reverse edgetics concerns the systematic separation of edgetic alleles that code for a protein defect (Charloteaux et al., 2011). These novel methods can differentiate between edgetic disruptions and node-removal mechanisms of phenotypic changes and pathogen infections. Edge direction is also essential to biological signaling systems/mechanisms and recent technology allows for the development of experimental methods to measure edgetic properties (Barzel and Barabasi, 2013). Continued exploration/experimentation will produce interactome network models on the proteome level that can integrate properties of edge strength, direction, and dynamics. Future interactome maps will combine weighted and animated edgetic information (Carvunis et al., 2013). Clearly, a database of all possible protein interactions for each species will be the next milestone in systems research.

## CONCLUSION

In summary, emerging technologies, resources, and research offer new opportunities to investigate unchartered territories in plant biology. Current interactome maps primarily reflect static states of time, internal conditions, and external influences. As such, today’s interactome maps should be utilized as a scaffold to model *in vivo* conditions by coalescing other layers of functional “-omic” data, including: genomics, phenomics, transcriptomics, metabolomics, and epigenomics. Integrating diverse plant “-omics” data enables researchers to investigate and address plant processes and responses, such as development, signal transduction pathways, RNA processing, protein modifications, cell cycle, and plant immune responses. A global understanding of plant stress and disease responses and phenotypic diversity will promote investigations of network topological properties. Computational tools, databases, and other systems resources will continue to grow and facilitate functional analysis and integration of multiple heterogeneous data sources. This may lead to improvements in environmental resilience, pathogen resistance, and overall crop production.

## AUTHOR CONTRIBUTIONS

Cassandra C. Garbutt, Purushotham V. Bangalore, Pegah Kannar, and M. S. Mukhtar wrote the manuscript. Cassandra C. Garbutt and M. S. Mukhtar prepared the figures. M. S. Mukhtar prepared the manuscript outline and coordinated the contributions from all co-authors.

## Conflict of Interest Statement

The authors declare that the research was conducted in the absence of any commercial or financial relationships that could be construed as a potential conflict of interest.
